# Dupilumab treatment for Chinese Nagashima-type palmoplantar keratoderma associated with atopic dermatitis: a case report

**DOI:** 10.3389/fimmu.2025.1647441

**Published:** 2025-09-25

**Authors:** Chunting Hua, Hao Cheng, Xianzhen Chen

**Affiliations:** Department of Dermatology, Sir Run Run Shaw Hospital, School of Medicine, Zhejiang University, Hangzhou, China

**Keywords:** Chinese Nagashima-type palmoplantar keratoderma, atopic dermatitis, dupilumab, SERPINB7, FLG

## Abstract

Patients with Nagashima-type palmoplantar keratoderma (NPPK) experience progressive, painful hyperkeratosis and fissuring of palms and soles that limits daily activities Due to the incomplete understanding of its pathogenesis, there are currently no effective treatments for NPPK. We report a 26-year-old woman with lifelong, worsening palmoplantar keratoderma, nail dystrophy, and concomitant atopic dermatitis refractory to topical treatments. Next-generation sequencing revealed compound heterozygous mutations in *SERPINB7* (c.796C>T, p.Arg266Ter) and *filaggrin* (FLG, c.3321delA, p.Gly1109GlufsTer13), while her asymptomatic parents and brother carried only single heterozygous variants, underscoring the digenic pathogenesis in our patient. After 42 weeks of dupilumab treatment, palmoplantar keratosis and nail changes had almost completely resolved, and the eruption resembled mild chronic eczema. Dupilumab therefore appears to be a safe and effective option for digenic NPPK complicated by atopic dermatitis and warrants further investigation in larger cohorts.

## Introduction

Nagashima-type palmoplantar keratoderma (NPPK) is an autosomal recessive genetic disorder caused by homozygous or compound heterozygous mutations in the *SERPINB7* (1, 2). It is clinically characterized by sharply demarcated erythema with mild-to-moderate hyperkeratosis on the palms and soles, often extending onto the dorsal aspects of the hands and feet. Patients may additionally develop concomitant fungal infections and eczematous lesions (3). Filaggrin (FLG), a key component of the cutaneous barrier, predisposes to atopic dermatitis when mutated, with *FLG* loss-of-function variants representing the strongest known genetic risk factor for the disease (4). Study identified a pathogenic *SERPINB7* variant as a risk factor for atopic dermatitis (AD) development, indicating that AD and NPPK may share an underlying pathogenic mechanism (5).

Current management of NPPK is hindered by several unresolved challenges. The efficacy of topical medication such as salicylic acid, urea, and α-hydroxy acids, is modest and usually transient, failing to achieve sustained control. Systemic retinoids are limited by dose-dependent toxicities including mucocutaneous dryness, hyperlipidemia and teratogenicity, necessitating rigorous monitoring (6). Recent study has demonstrated that topical gentamicin can significantly ameliorate childhood-onset hyperkeratosis, erythema, maceration, and desquamation (7). The benefit is restricted to patients with nonsense mutations, and chronic topical gentamicin may foster bacterial resistance; therefore, its potential for inducing antimicrobial resistance warrants further investigation. No mechanism-based or gene-targeted therapy has yet reached the clinic, leaving an unmet therapeutic need for patients whose pain, fissuring, and recurrent infections severely erode quality of life.

## Case presentation

**Figure 1:**
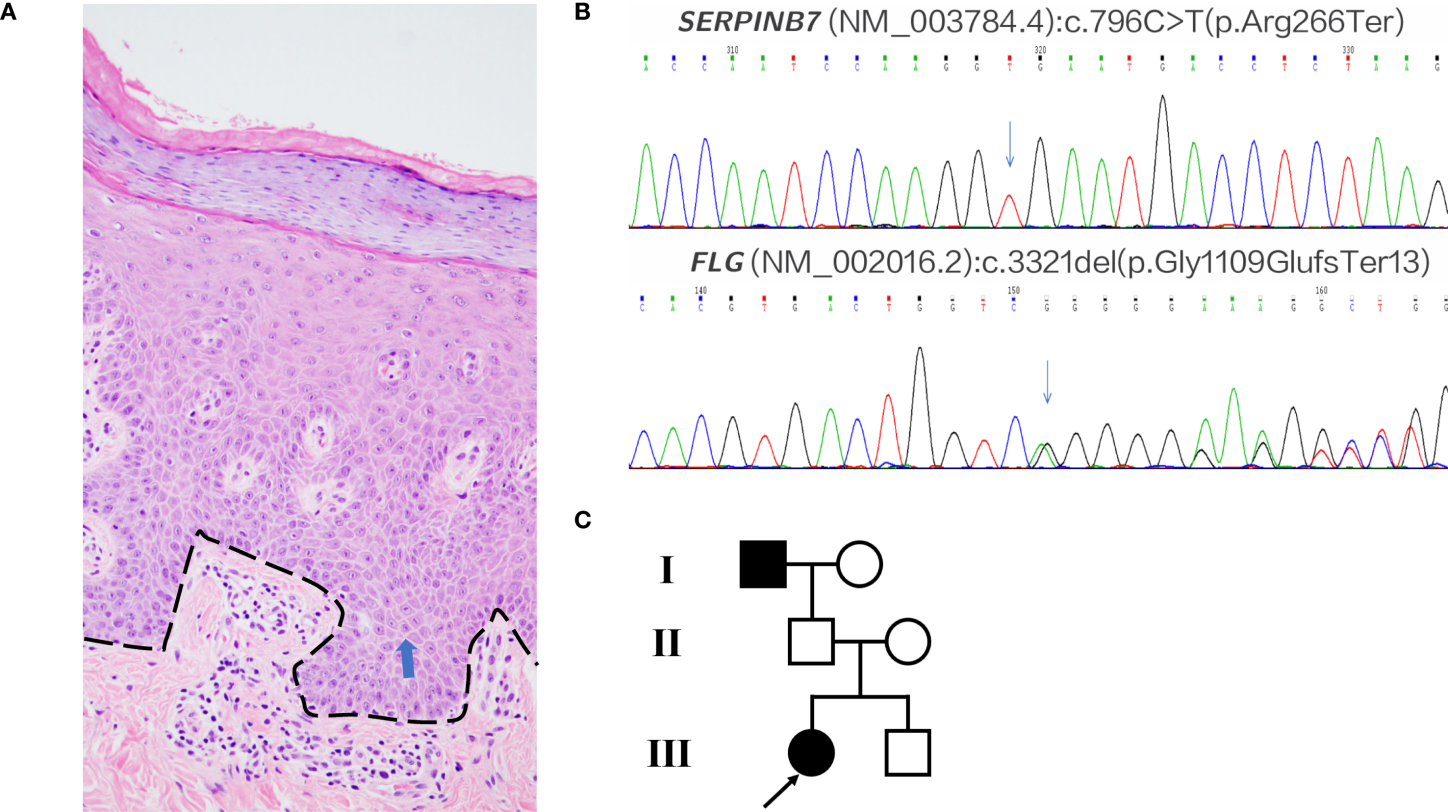
Histopathology and genetics. **(A)** Palm biopsy (H&E); dashed black line marks the dermal–epidermal junction; blue arrows indicate mild spongiosis. Family biopsies were declined. **(B)** Patient’s pathogenic variants: SERPINB7 c.796C>T and FLG c.3321delA. **(C)** Pedigree. Mother: FLG c.3321delA (het). Father: SERPINB7 c.796C>T (het). Brother: both variants (compound het).

A 26-year-old woman presented to our outpatient clinic with noticeable keratosis on the both palms, erythematous papules on the lateral hand edge, and occasional itching and tightness. She reported a history of erythema and keratosis on both palms, wrist flexures, thenar eminences, soles, and foot edges since childhood, which had gradually worsened over time. Previous topical treatments had alleviated keratosis and fissuring, but recent control of the rash was poor. Additionally, she noted gradual thickening, desquamation, and pruritus of the right fingernail over the past two years. On examination, well-demarcated keratotic erythema with marked hyperkeratosis and desquamation were observed in the aforementioned areas. Fungal examination of the fingernail was negative, but partial nail fissuring and dystrophy were present. The patient also had a history of atopic dermatitis for over a decade. Her IgE and eosinophil levels were within normal limits, and allergen testing revealed only a mild reaction to dust mites. Histopathological examination of the patient’s right palm during the treatment course revealed hyperkeratosis with parakeratosis, acanthosis, mild spongiosis, and perivascular mononuclear lymphocytic infiltration in the superficial dermis (**Figure 1A**). She was subsequently diagnosed with Nagashima-type Palmoplantar Keratoderma and atopic dermatitis.

## Treatment

Given the rare co-occurrence of Nagashima-type palmoplantar keratoderma and severe atopic dermatitis in the patient, we performed Next-Generation Sequencing. The analysis revealed a *SERPINB7* gene mutation (c.796C>T, p.Arg266Ter) and a *Filaggrin* (FLG) gene mutation (c.3321delA, p.Gly1109GlufsTer13) (**Figure 1B**). Additionally, we conducted medical history interviews and genetic testing on the patient’s immediate family members. The results revealed that the patient’s mother only had a heterozygous *FLG* mutation, the father only had a heterozygous *SERPINB7* mutation while the younger brother had heterozygous mutations in both genes. None of these family members exhibited symptoms of palmoplantar keratoderma or atopic dermatitis. Notably, the patient reported a similar history of palmoplantar keratoderma in her deceased grandfather, though no genetic testing was performed (**Figure 1C**).

**Figure 2:**
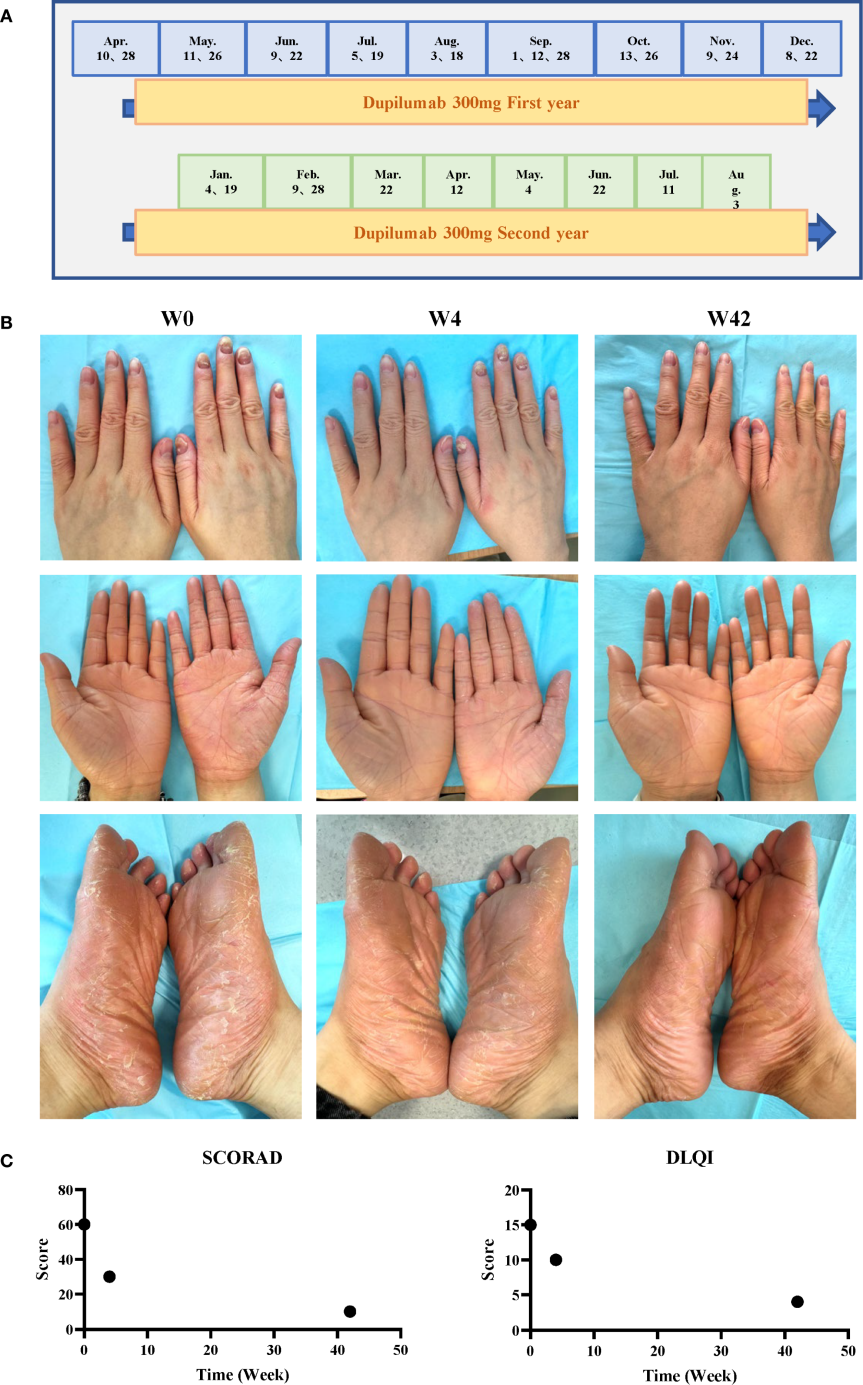
Treatment timeline and clinical outcomes. **(A)** Dupilumab dosing schedule. **(B)** Photographs obtained at baseline (week 0), after 4 doses (week 8), and after 22 doses (week 42). **(C)** Disease severity scores over time.

The patient had previously received upadacitinib 15 mg orally once daily but showed an inadequate response. Dupilumab therapy was initiated at a dose of 300 mg subcutaneously on April 10, 2024, followed by a maintenance dosing schedule of 300 mg every two weeks. The keratosis on palms and soles of the patient has shown significant improvement and fingernails essentially returned to normal state after dupilumab treatment (**Figure 2**).

## Discussion

NPPK is most prevalent among Asians, with estimated prevalences of 3.1 per 10,000 and 1.2 per 10,000 in the respective populations. Four recurrent SERPINB7 mutations—c.796C>T, c.522dupT, c.650_653delCTGT, and c.455G>T—account for 97.6% of all mutant alleles in Chinese patients, and the c.796C>T variant represents the founder mutation in Chinese and Japanese cohorts (3, 8). Outside Asia, NPPK is exceptionally rare; the first three Finnish cases were reported in 2020, carrying the homozygous founder mutation c.1136G>A (9). In 2023, four U.S. cases revealed novel variants c.806_818delinsT and c.828dup, presenting with painful, pruritic, malodorous fissures and hyperhidrosis; approximately 75% had comorbid atopic dermatitis (10).

The keratinization process and barrier function of the skin are interrelated. The abnormal hyperkeratosis in NPPK may potentially affect the integrity of the skin barrier, increasing the risk of atopic dermatitis. And *FLG* mutation c.3321delA has been reported in several patients with ichthyosis vulgaris and atopic dermatitis (11). From a genetic perspective, there may be some common regulatory genes or pathways between the two diseases that have not yet been clarified. To date, it’s the first reported case of combined *SERPINB7* and *FLG* gene mutations.

Dupilumab works by targeting and binding to IL-4Rα and preventing IL-4 and IL-13 signaling. It has been approved for several allergic and inflammatory indications and is also used off-label for a variety of conditions such as bullous pemphigoid (12), Netherton syndrome (13) and Hailey-Hailey disease (14, 15). These off-label uses of dupilumab highlight its potential versatility in treating a range of conditions beyond its approved indications. Three considerations prompted its off-label use in our patient. Firstly, the patient had failed all conventional topical and systemic therapy and there is no targeted therapy approved for NPPK. Secondly, dupilumab has recently been reported to effectively improve bullous pemphigoid with hyperkeratosis and palmoplantar keratoderma (16), suggesting benefit in disorders of abnormal keratinisation driven by IL-4/IL-13 hyperactivity. Thirdly, the patient had atopic dermatitis that met standard prescribing criteria, making dupilumab a rational, dual-purpose therapeutic choice.

Over one year of follow-up, dupilumab markedly improved palmoplantar keratoderma in our patient with NPPK, without relapse or adverse events. These encouraging findings are tempered by the inherent limitations of a single-patient report: limited generalizability, absence of controls, and lack of validated biomarkers for objective quantification of response. Future multi-center, placebo-controlled trials with standardized clinical and molecular endpoints are essential to confirm efficacy, define optimal dosing, and elucidate the precise mechanisms through which IL-4/IL-13 inhibition benefits NPPK.

## Data Availability

The original contributions presented in the study are included in the article/supplementary material. Further inquiries can be directed to the corresponding authors.
